# Supplementary material for: C2CAplus: A One-Pot Isothermal Circle-to-Circle DNA Amplification System
Source: ACS Synth Biol. 2023 Sep 20;12(10):3137–42. doi: 10.1021/acssynbio.3c00390 (PMC10594867; doi:10.1021/acssynbio.3c00390)
Supplement: Supplementary file 1 — sb3c00390_si_001.pdf [file sb3c00390_si_001.pdf]

C2CAplus: a one-pot isothermal circle-to-circle DNA amplification system

Laura Grasemann, Paula Thiel Pizarro, and Sebastian J. Maerkl*

Institute of Bioengineering, School of Engineering, École Polytechnique Fédérale de Lausanne, 1015 Lausanne, Switzerland

August 16, 2023

Supplementary Information

*Correspondence: sebastian.maerkl@epfl.ch

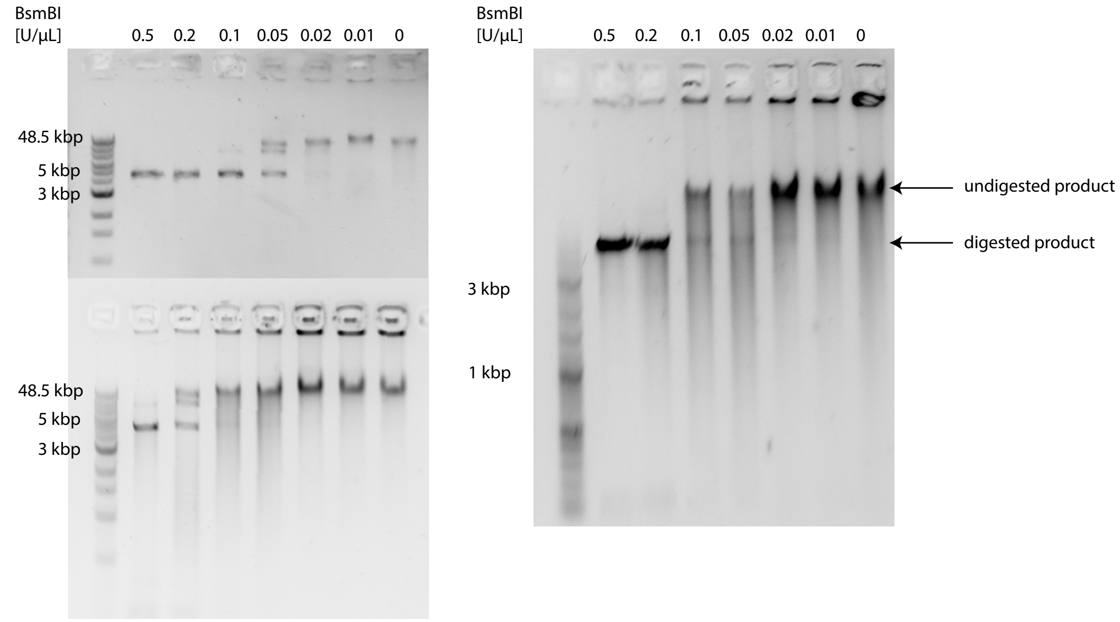

Figure 1: BsmBI titration at 30°C and 18 h reaction time. Three independent replicates were performed. A concentration of 0.5 to 0.2 U/µL digests most of the RCA product in the given conditions.

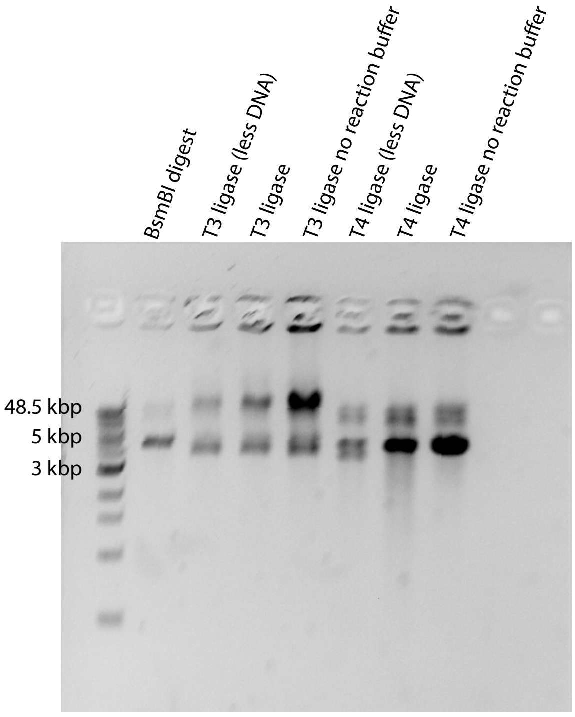

Figure 2: Non-cropped gel of Figure 1C. The first lane is a BsmBI digested product as shown in Figure 1C. The second lane is a T3 ligase ligation reaction with less DNA (3 µL in a total volume of 10 µL), not included in Figure 1C. The following two lanes are again included in Figure 1C, and show the T3 ligation with and without reaction buffer. The 5th lane, T4 ligase (less DNA) is not included in Figure 1C, and shows the T4 ligation with 3 µL of DNA in a total volume of 10 µL. The last two lanes are again included in Figure 1C, and show the T4 ligation reaction with and without reaction buffer.

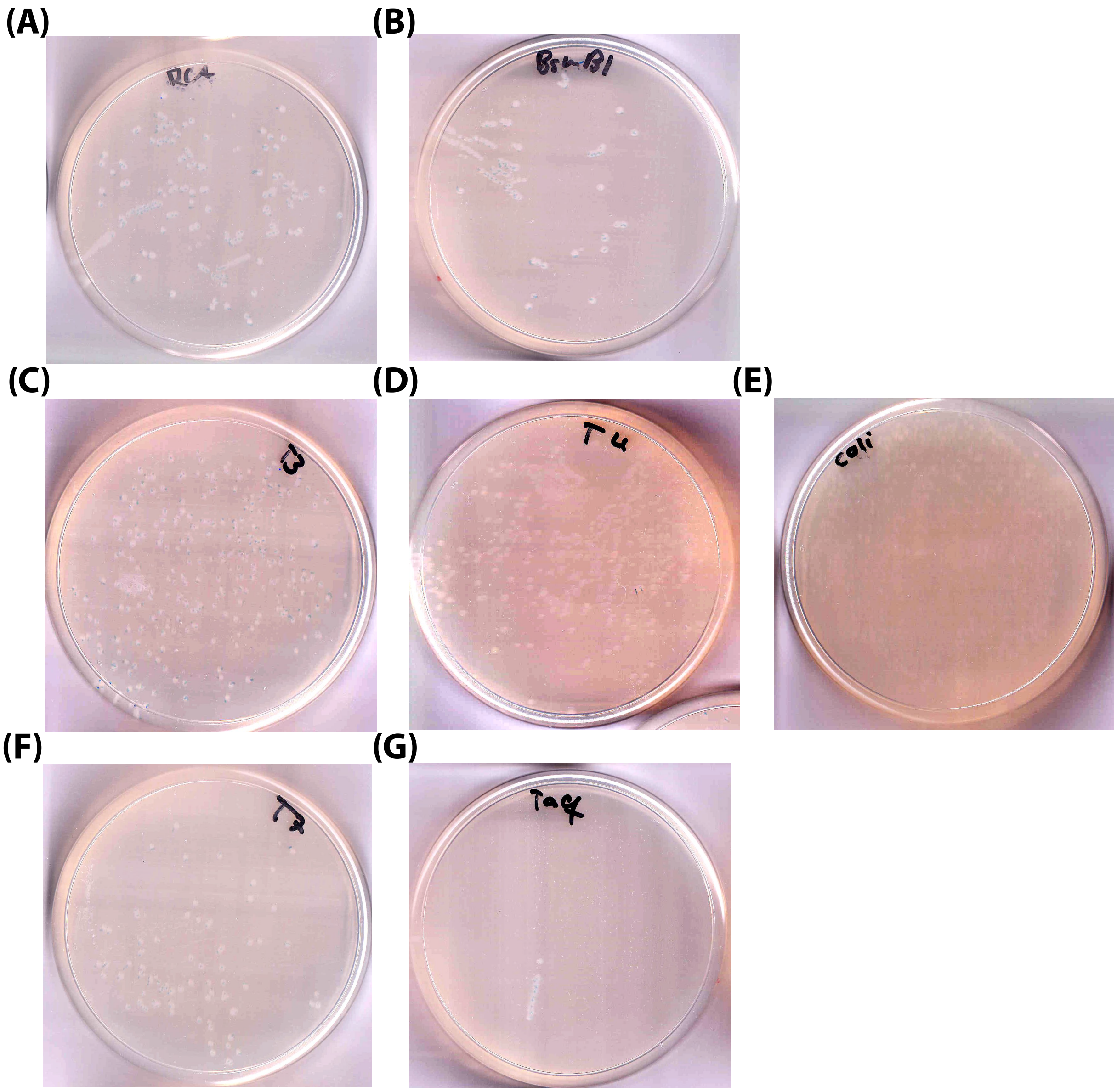

Figure 3: Ligase screen. Transformation results of: (A) non-treated RCA product, (B) BsmBI digested RCA product, T3 (C) T3 ligase, T4 (D) T4 ligase, (E) *E. coli* ligase, (F) T7 ligase, (G) Taq ligase.

DNA sequences

Primer sequences

The primers used in this work were:

Table 1: Primers used during this work

5′ final	GATCTTAAGGCTAGAGTAC
3′ final	CAAAAAACCCCTCAAGAC

Plasmid sequence

The plasmid used in this work is a pSTBlue plasmid encoding Kanamycin and Ampicillin resistance as well as an EGFP gene. The BsmBI restriction site is located inside the Kanamycin resistance gene. The complete plasmid sequence is:

CTCAGGCGCAATCACGAATGAATAACGGTTTGGTTGATGCGAGTGATTTTGATGACG
AGCGTAATGGCTGGCCTGTTGAACAAGTCTGGAAAGAAATGCATAAACTTTTGCCATTC
TCACCGGATTCAGTCGTCACTCATGGTGATTTCTCACTTGATAACCTTATTTTTGACGAGG
GGAAATTAATAGGTTGTATTGATGTTGGACGAGTCGGAATCGCAGACCGATACCAGGAT
CTTGCCATCCTATGGAACTGCCTCGGTGAGTTTTCTCCTTCATTACAGAAACGGCTTTTTC
AAAAATATGGTATTGATAATCCTGATATGAATAAATTGCAGTTTCATTTGATGCTCGATG
AGTTTTTCTAAGAATTAATTCATGACCAAAATCCCTTAACGTGAGTTTTCGTTCCACTGAG
CGTCAGACCCCGTAGAAAAGATCAAAGGATCTTCTTGAGATCCTTTTTTTCTGCGCGTAA
TCTGCTGCTTGCAAACAAAAAAACCACCGCTACCAGCGGTGGTTTGTTTGCCGGATCAA
GAGCTACCAACTCTTTTTCCGAAGGTAACTGGCTTCAGCAGAGCGCAGATACCAAATAC
TGTCCTTCTAGTGTAGCCGTAGTTAGGCCACCACTTCAAGAACTCTGTAGCACCGCCTAC
ATACCTCGCTCTGCTAATCCTGTTACCAGTGGCTGCTGCCAGTGGCGATAAGTCGTGTCT
TACCGGGTTGGACTCAAGACGATAGTTACCGGATAAGGCGCAGCGGTCGGGCTGAACG
GGGGGTTCGTGCACACAGCCCAGCTTGGAGCGAACGACCTACACCGAACTGAGATACC
TACAGCGTGAGCTATGAGAAAGCGCCACGCTTCCCGAAGGGAGAAAGGCGGACAGGTA
TCCGGTAAGCGGCAGGGTCGGAACAGGAGAGCGCACGAGGGAGCTTCCAGGGGGAAA

CGCCTGGTATCTTTATAGTCCTGTCGGGTTTCGCCACCTCTGACTTGAGCGTCGATTTTTG
TGATGCTCGTCAGGGGGGCGGAGCCTATGGAAAAACGCCAGCAACGCGGCCTTTTTACG
GTTCCTGGCCTTTTGCTGGCCTTTTGCTCACATGTTCTTTCCTGCGTTATCCCCTGATTCTG
TGGATAACCGTATTACCGCCTTTGAGTGAGCTGATACCGCTCGCCGCAGCCGAACGACC
GAGCGCAGCGAGTCAGTGAGCGAGGAAGCGGAAGAGCGCCCAATACGCAAACCGCCT
CTCCCCGCGCGTTGGCCGATTCATTAATGCAGCTGGCACGACAGGTTTCCCGACTGGAA
AGCGGGCAGTGAGCGCAACGCAATTAATGTGAGTTAGCTCACTCATTAGGCACCCCAGG
CTTTACACTTTATGCTTCCGGCTCGTATGTTGTGTGGAATTGTGAGCGGATAACAATTTCA
CACAGGAAACAGCTATGACCATGATTACGCCAAGCTCTAATACGACTCACTATAGGGAA
AGCTCGGTACCACGCATGCTGCAGACGCGTTACGTATCGGATCCAGAATTCGTGATGAT
CTTAAGGCTAGAGTACTAATACGACTCACTATAGGGAGACCACAACGGTTTCCCTCTAG
AAATAATTTTGTTTAACTTAAGAAGGAGGAAAAAAAAATGTCTAAAGGTGAAGAATTAT
TCACTGGTGTTGTCCCAATTTTGGTTGAATTAGATGGTGATGTTAATGGTCACAAATTTTC
TGTCTCCGGTGAAGGTGAAGGTGATGCTACTTACGGTAAATTGACCTTAAAATTTATTTG
TACTACTGGTAAATTGCCAGTTCCATGGCCAACCTTAGTCACTACTTTAACTTATGGTGTT
CAATGTTTTTCTAGATACCCAGATCATATGAAACAACATGACTTTTTCAAGTCTGCCATG
CCAGAAGGTTATGTTCAAGAAAGAACTATTTTTTTCAAAGATGACGGTAACTACAAGAC
CAGAGCTGAAGTCAAGTTTGAAGGTGATACCTTAGTTAATAGAATCGAATTAAAAGGTA
TTGATTTTAAAGAAGATGGTAACATTTTAGGTCACAAATTGGAATACAACTATAACTCTC
ACAATGTTTACATCATGGCTGACAAACAAAAGAATGGTATCAAAGTTAACTTCAAAATT
AGACACAACATTGAAGATGGTTCTGTTCAATTAGCTGACCATTATCAACAAAATACTCC
AATTGGTGATGGTCCAGTCTTGTTACCAGACAACCATTACTTATCCACTCAATCTGCCTT
ATCCAAAGATCCAAACGAAAAGAGAGACCACATGGTCTTGTTAGAATTTGTTACTGCTG
CTGGTATTACCCATGGTATGGATGAATTGTACAAATAATAACGACTCAGGCTGCTACGC
CTGTGTACTGGAAAACAAAACCAAAACCCAAAAAACAAAAAACTGAGCCCATTGGTAT
CGTGGAAGGACTCTATCAAAAAAAAAAAAAAAAAAAAAAAAAAAACTAGCATAAC
CCCTTGGGGCCTCTAAACGGGTCTTGAGGGGTTTTTTGATCTGAATTCGTCGACAAGCTT
CTCGAGCCTAGGCTAGCTCTAGACCACACGTGTGGGGGCCCGAGCTCGCGGCCGCTGTA
TTCTATAGTGTCACCTAAATGGCCGCACAATTCACTGGCCGTCGTTTTACAACGTCGTGA
CTGGGAAAACCCTGGCGTTACCCAACTTAATCGCCTTGCAGCACATCCCCCTTTCGCCAG
CTGGCGTAATAGCGAAGAGGCCCGCACCGATCGCCCTTCCCAACAGTTGCGCAGCCTGA

ATGGCGAATGGAAATTGTAAGCGTTAATATTTTGTTAAAATTCGCGTTAAATTTTTGTTAA
ATCAGCTCATTTTTTAACCAATAGGCCGAAATCGGCAAAATCCCTTATAAATCAAAAGA
ATAGACCGAGATAGGGTTGAGTGTTGTTCCAGTTTGGAACAAGAGTCCACTATTAAAGA
ACGTGGACTCCAACGTCAAAGGGCGAAAAACCGTCTATCAGGGCGATGGCCCACTACG
TGAACCATCACCCTAATCAAGTTTTTTGGGGTCGAGGTGCCGTAAAGCACTAAATCGGA
ACCCTAAAGGGAGCCCCCGATTTAGAGCTTGACGGGGAAAGCCGGCGAACGTGGCGAG
AAAGGAAGGGAAGAAAGCGAAAGGAGCGGGCGCTAGGGCGCTGGCAAGTGTAGCGGT
CACGCTGCGCGTAACCACCACACCCGCCGCGCTTAATGCGCCGCTACAGGGCGCGTCAG
GTGGCACTTTTCGGGGAAATGTGCGCGGAACCCCTATTTGTTTATTTTTCTAAATACATTC
AAATATGTATCCGCTCATGAGACAATAACCCTGATAAATGCTTCAATAATATTGAAAAA
GGAAGAGTATGAGTATTCAACATTTCCGTGTCGCCCTTATTCCCTTTTTTGCGGCATTTTG
CCTTCCTGTTTTTGCTCACCCAGAAACGCTGGTGAAAGTAAAAGATGCTGAAGATCAGTT
GGGTGCACGAGTGGGTTACATCGAACTGGATCTCAACAGCGGTAAGATCCTTGAGAGTT
TTCGCCCCGAAGAACGTTTTCCAATGATGAGCACTTTTAAAGTTCTGCTATGTGGCGCGG
TATTATCCCGTATTGACGCCGGGCAAGAGCAACTCGGTCGCCGCATACACTATTCTCAG
AATGACTTGGTTGAGTACTCACCAGTCACAGAAAAGCATCTTACGGATGGCATGACAGT
AAGAGAATTATGCAGTGCTGCCATAACCATGAGTGATAACACTGCGGCCAACTTACTTC
TGACAACGATCGGAGGACCGAAGGAGCTAACCGCTTTTTTGCACAACATGGGGGATCAT
GTAACTCGCCTTGATCGTTGGGAACCGGAGCTGAATGAAGCCATACCAAACGACGAGC
GTGACACCACGATGCCTGTAGCAATGGCAACAACGTTGCGCAAACTATTAACTGGCGAA
CTACTTACTCTAGCTTCCCGGCAACAATTAATAGACTGGATGGAGGCGGATAAAGTTGC
AGGACCACTTCTGCGCTCGGCCCTTCCGGCTGGCTGGTTTATTGCTGATAAATCTGGAGC
CGGTGAGCGTGGGTCTCGCGGTATCATTGCAGCACTGGGGCCAGATGGTAAGCCCTCCC
GTATCGTAGTTATCTACACGACGGGGAGTCAGGCAACTATGGATGAACGAAATAGACA
GATCGCTGAGATAGGTGCCTCACTGATTAAGCATTGGTAACTGTCAGACCAAGTTTACTC
ATATATACTTTAGATTGATTTAAAACTTCATTTTTAATTTAAAAGGATCTAGGTGAAGATC
CTTTTTGATAATCTCATGAACAATAAAACTGTCTGCTTACATAAACAGTAATACAAGGGG
TGTTATGAGCCATATTCAACGGGAAACGTCTTGCTCTAGGCCGCGATTAAATTCCAACAT
GGATGCTGATTTATATGGGTATAAATGGGCTCGCGATAATGTCGGGCAATCAGGTGCGA
CAATCTATCGATTGTATGGGAAGCCCGATGCGCCAGAGTTGTTTCTGAAACATGGCAAA
GGTAGCGTTGCCAATGATGTTACAGATGAGATGGTCAGACTAAACTGGCTGACGGAATT

TATGCCTCTTCCGACCATCAAGCATTTTATCCGTACTCCTGATGATGCATGGTTACTCACC
ACTGCGATCCCCGGGAAAACAGCATTCCAGGTATTAGAAGAATATCCTGATTCAGGTGA
AAATATTGTTGATGCGCTGGCAGTGTTCCTGCGCCGGTTGCATTCGATTCCTGTTTGTAAT
TGTCCTTTTAACAGCGATCGCGTATTTCGTCTCG
